# Correlation of *TCF4, GSK, TERT* and *TERC* Expressions with Proliferation Potential of Early and Late Culture of Human Peripheral Blood Mesenchymal Stem Cells 

**DOI:** 10.22074/cellj.2021.6920

**Published:** 2020-04-22

**Authors:** Zahra Fazeli, Masoumeh Rajabibazl, Sepideh Faramarzi, Mir Davood Omrani, Sayyed Mohammad Hossein Ghaderian, Niloufar Safavi Naini

**Affiliations:** 1. Department of Medical Genetics, Faculty of Medicine, Shahid Beheshti University of Medical Sciences, Tehran, Iran; 2. Department of Clinical Biochemistry, Faculty of Medicine, Shahid Beheshti University of Medical Sciences, Tehran, Iran

**Keywords:** Cellular Senescence, Mesenchymal Stem Cells, Regenerative Medicine, WNT Signaling Pathway

## Abstract

**Objective:**

In the recent years, mesenchymal stem cells (MSCs) were considered as the suitable source of cells for transplantation into the damaged tissues in regenerative medicine. There was low number of these cells in different organs and this characteristic was the main drawback to use them in treatment of diseases. Cellular senescence of the stem cells has been demonstrated to be dependent to the telomerase activity. The aim of present experimental study was to evaluate correlation of the expression of telomerase components and WNT signaling pathway in MSCs derived from human peripheral blood (PB-MSCs).

**Materials and Methods:**

In this experimental study, following the isolation of MSCs from peripheral blood mononuclear cells, RNA was extracted from these cells in the early culture (8-9^th^ days) and late culture (14-17^th^ days). Then, expression of* TERT, TERC, TCF4, GSK* and *CTNNB1* was determined by quantitative reverse transcription polymerase chain reaction (qRT-PCR) based on SYBR Green.

**Results:**

Our data indicated that there was a significantly reduced expression of *TERT* in the late culture of human MSCs derived from peripheral blood (P<0.05). Although a negative correlation was observed between GSK and TERC expression levels in the early culture of MSCs, spearman analysis showed that there was no significant correlation between the expression of telomerase components (*TERC* and *TERT*) and WNT signaling pathway (P>0.05).

**Conclusion:**

The obtained results suggested that WNT signaling pathway likely plays a minor role in the maintenance of telomere length and proliferation potential of MSCs.

## Introduction

Following the characterization of self-renewal and differentiation abilities of mesenchymal stem cells (MSCs), these cells were considered as suitable candidates in the field of tissue engineering and repair of damaged tissues (1). MSCs have been demonstrated to be isolated from different sources including bone marrow, synovium, umbilical cord, adipose tissue and peripheral blood (2, 3). To use MSCs in cell therapy, it is necessary to obtain enough number of MSCs following the long-term culture of these cells. However, the prolonged culture was associated with cellular senescence (4). Identification of the mechanisms regulating MSC senescence could play a key function in preventing the aging in these cells.

Telomere length has been revealed to play an important role in the cellular senescence. Maintaining telomere length by telomerase prevented arrest of cell proliferation (5, 6). Izadpanah et al. (7) demonstrated presence of telomerase activity (TA) in MSCs. Their results indicated that TA was decreased with aging at MSCs. The constitutive expression of telomerase was accompanied with the enhanced proliferation ability of MSCs without any side-effect on their differentiation potential (8). Different studies showed that TA was dependent to the human telomerase reverse transcriptase (*TERT*) expression. TERT expression was regulated mainly at transcription level (9).

Analysis of the MSC expression profile has revealed that several signaling pathways, including WNT signaling, play role in different biological treats (10, 11). WNT signaling pathway has been demonstrated to be involved in several cellular processes including stem cell renewal (12). Following the interaction of WNT with its receptor, the corresponding signal was transduced to the downstream molecule, known as Dsh. This transduction led to the disruption of APC/Axin/ GSK3 complex. This event prevented degradation of β-catenin. After translocation of β-catenin from the cytoplasm into the nucleus, this protein formed a complex with TCF4 and then, this complex trigger transcription of the target genes (12, 13). Zhang et al. (14) reported that WNT signaling had an ability to regulate TERT expression in cancer and somatic cells. They demonstrated that knockdown of β-catenin by shRNA led to TA decrease in cancer cells.

In the study performed by Gry et al. (15), correlation of RNA level with protein was evaluated for different genes. Their results indicated significant correlation of the RNA with protein level in 33% of the cases. The aim of present study was to investigate whether RNA expression of *TERT* and telomerase RNA component (TERC) depend on expression of the WNT signaling pathway genes in the early and late culture of MSCs derived from peripheral blood (PB-MSCs). This finding could increase our understanding about the molecular mechanisms of MSC cellular senescence.

## Materials and Methods

### Preparation of human mesenchymal stem cells derived from peripheral blood

In this experimental study, 20 ml peripheral blood was collected from three females aged 35-40 years. The Ficoll density gradient method was used to isolate mononuclear cells from the collected human peripheral blood as previously described (16). The obtained cell pellet was cultured in Dulbecco’s Modified Eagle Medium: Nutrient Mixture F-12 medium (DMEM-F12, BioIdea, Iran) including 10% fetal bovine serum (FBS, Gibco, USA), 2 mM L-Glutamate (BioIdea, Iran) and 100 U/ml penicillin/streptomycin (Gibco, Germany). After 72 hours, the medium containing non-adherent cells was replaced with the fresh medium. Growth of the cells was monitored under an inverted microscope. The culture cells were usually reached 70-80% confluence after six days (D6). Phenotypic characterization of these cells was confirmed as MSCs by flow cytometry with CyFlow Space (Partec, Germany). This study was performed on MSC cultures after 8-9 days and 14- 17 days; they were known as early and late culture, respectively. MSC culture on day 6th was used as control.

### Quantitative reverse transcription polymerase chain reaction

Total RNA purification kit (Jena Bioscience, Germany) was used to obtain Total RNA from the cultured cells. In the next step, DNase I (Fermentas, USA) treatment was performed to remove DNA contamination. After that, RevertAid First Strand cDNA Synthesis kit (Thermo Scientific, USA) was used to synthesize cDNA. Next, quantitative reverse transcription polymerase chain reaction (qRT-PCR) was carried out in duplicate using RealQ Plus Master Mix Green (Ampliqon, Denmark). Condition of the reaction was performed as follow: 95˚C for 10 minutes followed by 40 cycles of denaturation at 95˚C for 30 seconds, annealing at 60˚C for 30 seconds, and extension at 72˚C for 30 seconds. The sequences of primer sets are presented in Table 1. Specificity of qRT-PCR products was confirmed by melting curve analysis as well as the electrophoresis of 1.5% agarose gel (Genfanavaran, Iran) stained with Safe stain (Yekta Tajhiz Azma, Iran).

### Compliance with ethical standards

All procedures performed in this study including human participants were in accordance with the ethical standards of the institutional and/or national research committee and with the 1964 Helsinki declaration and the relative later amendments or comparable ethical standards. The present study was approved by the Ethics Committee of the School of Medicine Shahid Beheshti University of Medical Sciences (Tehran, Iran, Ethical code: IR.SBMU.MSP.REC.1397.550). The manuscript have been read and confirmed by all authors.

**Table 1:** The sequence of primers used in the present study


Gene symbol	Primer sequence (5ˊ-3ˊ)	Product length (bp)

*HSP90AB1*	F: GGAAGTGCACCATGGAGAGGA	157
	R: GCGAATCTTGTCCAAGGCATCAG	
*TERT*	F: GGAGCAAGTTGCAAAGCATTG	182
	R: TCCCACGACGTAGTACATGTT	
*TERC*	F: CTGGGAGGGGTGGTGGCCATTT	179
	R: CGAACGGGCCAGCAGCTGACAT	
*GSK3B*	F: TCGAGAGCTCCAGATCATGAGAA	124
	R: CGGAACATAGTCCAGCACCAGA	
*CTNNB1*	F: TCTGAGGACAAGCCACAAGATTACA	122
	R: TGGGCACCAATATCAAGTCCAA	
*TCF4*	F: GCACTGCCGACTACAATAGG	150
	R: CTGCATAGCCAGGCTGATTC	


### Statistical analysis

Relative expression level of the studied genes was estimated by using the pfaffl method. The present study was performed in three independent experiments and HSP90AB1 was used as the housekeeping gene to normalize the qRT-PCR data. Student’s t test was used to define difference between the early and late cultures of MSC. Correlation between the expression of telomerase components and WNT signaling pathway genes was defined by estimating the Spearman correlation coefficient (rs). A P<0.05 was considered statistically significant. These analyses were performed using Social Science Statistics website (<uri>http://www.socscistatistics. com/tests/studentttest/Default2.aspx</uri>).

## Results

**Fig.1:**
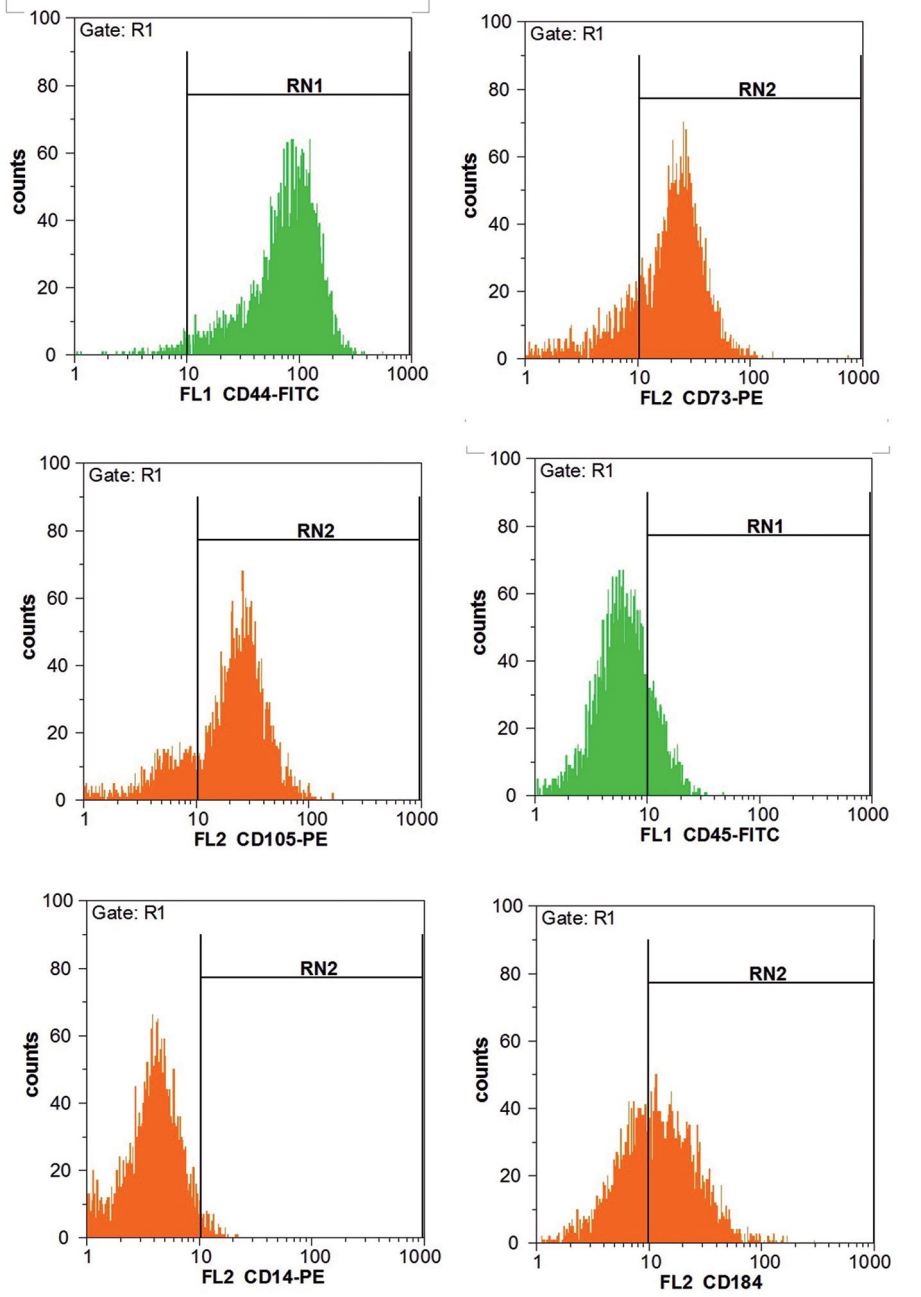
Results obtained from the flow cytometry analysis of peripheral blood-mesenchymal stem cells (PB-MSCs). CD marker expression analyses indicated that these cells were positive for CD184, CD105, CD73 and CD44, while they were negative for CD14 and CD45.

**Fig.2:**
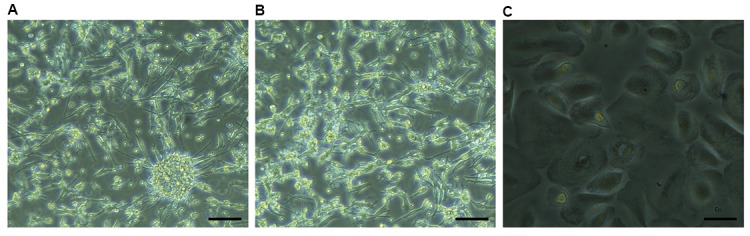
Morphology of peripheral blood-mesenchymal stem cells (PB-MSCs). **A.** These cells showed fibroblast like morphology at day 6^th^ of initial culture, **B.** Their morphology was changed over the time, and **C.** They were appeared flat and wide at the late culture (scale bar: 50 µm).

CD marker analysis of the stem cells in the present study indicated that these cells expressed CD184, CD105, CD73 and CD44. No expression was determined in these cells for CD14 and CD45 (Fig.1). Pattern of the surface markers on these cells confirmed identity of these cells as MSCs. These cells showed fibroblast like morphology at the day 6th of culture. Appearance of these cells was changed along with increasing the age. These cells showed flat and wide morphology under inverted microscope at the days 14^th^ -17^th^ of culture (Fig.2).

**Fig.3:**
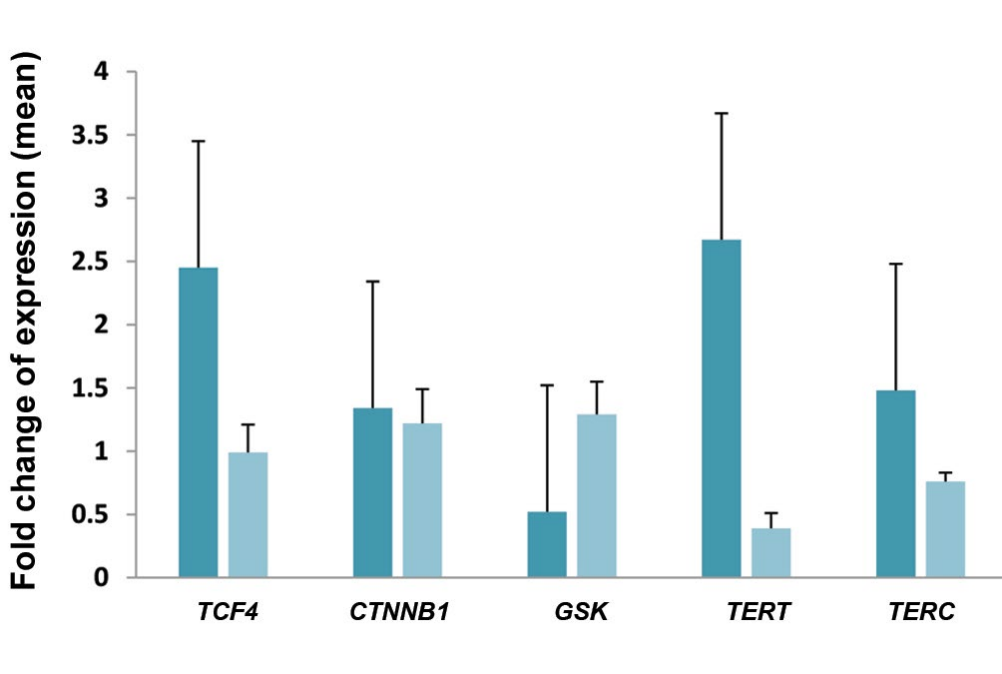
Mean expression levels of *TERT, TERC, TCF4, CTNNB1* and *GSK* in the early mesenchymal stem cells (MSC) culture compared to the late culture. Three independent experiments were performed and the expression levels were normalized to those of *HSP90AB1* gene.

Relative expression level analysis of the studied genes indicated a significant down-regulation of *TERT* in the late culture of MSCs (t test:-2.29, P=0.04, Table 2). The obtained results suggested that low expression of *TERT* and *TERC* in 14-17 days of the culture were accompanied with the diminished *TCF4* expression and enhanced GSK expression in 8-9 days of MSCs culture (Fig.3, Table 2). Furthermore, we found that enhanced expression of *TERC* was associated with the enhanced *CTNNB1* and diminished *GSK* expressions in the early MSCs culture (Table 2). Spearman analysis indicated that there was a weak correlation between TERC and CTNNB1, GSK as well as TCF4 expression. However, this correlation was not statistically significant (P>0.05, Table 3).

**Table 2:** The results obtained from Student’s t test (two-sided) analysis for the expression data of the studied genes in early and late mesenchymal stem cells culture


The studied genes	Early term culture		Late term culture	
	t test	P value	t test	P value

TERC	1.82	0.14	-1.43	0.18
*TERT*	0.17	0.87	-2.29	0.04^*^
*TCF4*	0.21	0.85	-0.64	0.54
*CTNNB1*	1.19	0.30	1.44	0.18
*GSK*	-1.22	0.29	0.92	0.38


*; Statistically significant.

**Table 3:** Relationship of telomerase component expressions with WNT signaling pathway using the Shearman correlation coefficient analysis


The WNT signaling pathway genes	Early term culture				Long term culture			
		TERC		TERT		TERC		TERT	
	r_s_	P value	r_s_	P value	r_s_	P value	r_s_	P value

TCF4	0.5	1	-0.5	1	0.143	0.803	-0.371	0.497
CTNNB1	-1	0.333	-0.5	1	0.486	0.355	-0.143	0.803
GSK	-0.5	1	0.5	1	0.257	0.658	-0.428	0.419


r_S_; Spearman correlation coefficient

## Discussion

In the recent years, MSC was considered as a favorable cellular model in treatment of different diseases. Safety and efficacy of these cells have been confirmed in many clinical trials performed by MSC administrations. However, understanding MSC biological characteristics improved application of these cells in clinic. Zhao et al. (17) showed that activity of telomerase was decreased in long-term culture of MSCs derived from bone marrow of Sprague Dawley rats. They also demonstrated that overexpression of TERT was associated with the enhanced proliferation rate and decreased MSC senescence.

In the present study, we found that expressions of TERT and TERC were decreased with the aging of human PB-MSCs, which was consistent with the results obtained from MSCs derived from human bone marrow and adipose tissue (18). TA has been demonstrated to be important in different characteristics of stem cell including proliferation and differentiation abilities. Kang et al. (19) showed that transfection of telomerase reverse transcriptase gene into MSCs could enhance life span and differentiation ability.

Some studies indicated that TERC expression could participate in up-regulation or down-regulation of the other genes including the genes involved in glycolytic pathway, angiogenesis and metastasis as well as NF-κB target genes (20-22). Although the results obtained from the spearman correlation analysis indicated that *TERT* expression did not show significant correlation with the expression of WNT signaling pathway genes in the PB-MSCs, we observed negative correlation of GSK with *TERT* expression in the studied cells. These results suggested possible function of TERT in the regulation of WNT signaling pathway genes.

Different studies demonstrated that β-catenin phosphorylation, through GSK, contributed to its degradation, resulting in the suppression of WNT signaling pathway (23). Association of the enhanced expression of TERC with the decreased expression of GSK in early culture of MSCs was supported by stabilization and activation of β-catenin in the early culture of MSCs. Furthermore, negative association of *TERC* with *GSK* expression (data not shown) suggested that TERC indirectly regulates activity of β-catenin gene, via GSK, in the early culture of MSCs.

There were several hypotheses about how telomerase could influence expression of the other genes, one of which proposed that telomerase enzyme influences gene expression through alteration of chromatin structure. The other hypothesis indicated that interaction of telomerase with different transcription factors coordinates in the gene transcriptional regulation (23-25). Expression of *TCF4* and *TERT* supported the impact of TERT interaction with TCF4 on the expression of down-stream WNT signaling pathway genes.

## Conclusion

Several pathways including WNT signaling pathway have been revealed to be involved in telomerase regulation and self-renewal ability of the stem cells. However, there was no report about the effect of WNT signaling pathway on the expression of telomerase components in the MSCs derived from human PB-MSCs. Our data indicated that activation of WNT signaling in early culture of MSCs may contribute to the enhanced expression of *TERC* and *TERT*, while this signaling pathway appears to have a minor role in the expression of telomerase components and possibly telomerase activity. Taken together, these findings suggested that investigating other signaling pathways could improve our knowledge in the regulation of TERT and TERC.
